# Traceability of *Manilkara huberi* Using Stable Isotope Ratio Analysis to Support Deforestation Prevention in the Amazon

**DOI:** 10.1021/acsomega.6c03050

**Published:** 2026-09-22

**Authors:** Helinara L. V. Capucho, José H. de Oliveira, Ricardo Lopes, Santiago L. F. Ramos, Ananda V. de Aguiar, Carlos H. S. G. Meneses, Ricardo L. S. Marques, Fabio de C. Borba, Gabriela B. Nardoto, Maria T. G. Lopes

**Affiliations:** † Faculty of Agricultural Sciences, 67892 Federal University of Amazonas (UFAM), Manaus 69077-000, AM, Brazil; ‡ Technical-Scientific Sector, 383815 Federal Police (Brazil), Manaus 69042-470, AM, Brazil; § Brazilian Agricultural Research Corporation (Embrapa), Western Amazon Agroforestry Research Center, Manaus 69010-970, AM, Brazil; ∥ 548970 Brazilian Agricultural Research Corporation, Embrapa Floresta, Colombo 83411-000, PR, Brazil; ⊥ Department of Biology, State University of Paraíba (UEPB), Campina Grande 58429-500, PB, Brazil; # Institute of Biological Sciences, University of Brasília (UnB), Brasília 70910-900, DF, Brazil

## Abstract

Illegal deforestation represents one of the main challenges to the integrity of the Amazon rainforest, warranting the use of reliable tracking methods to identify the geographical origin of timber species inspected during commercial transactions. This study utilizes stable carbon (δ^13^C) and nitrogen (δ^15^N) isotopes to determine the origins of *Manilkara huberi* (massaranduba) and to establish a database of its geographical provenance. A total of 130 individual trees were sampled, yielding 350 wood samples, in the Brazilian states of Amazonas, Pará, Rondônia, and Roraima, and these wood samples were analyzed using isotope-ratio mass spectrometry. The results revealed significant differences in δ^13^C and δ^15^N isotopic signatures, which were associated with variations in soil properties, vegetation type, and local environmental conditions. Linear discriminant analysis yielded classification performance ranging from 14% to 97%, with the highest accuracies observed in specific municipalities. Nitrogen isotope ratios in the wood showed stronger geographic differentiation, whereas carbon isotope ratios reflected broader environmental influences. This study presents a georeferenced isotopic approach based on living trees, enabling Brazilian agencies to strengthen their use of this technique to combat illegal timber trade and to provide technical support for the evaluation and validation of Forest Origin Documents in Brazil.

## Graphical abstract

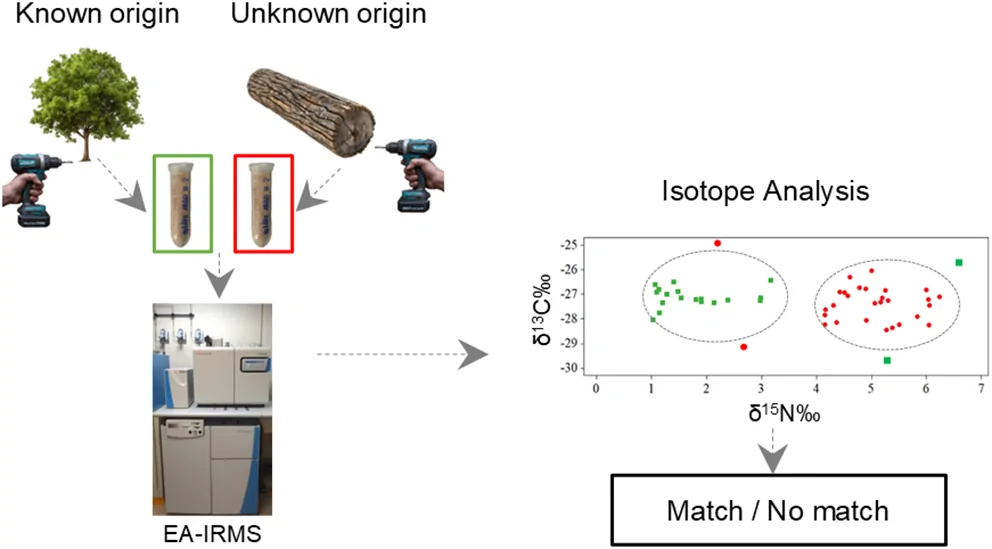

## 1 Introduction

The Amazon rainforest contains one of the highest levels of plant biodiversity worldwide and plays a central role in global climate regulation and biogeochemical cycles, particularly through carbon sequestration in tree biomass.^1–5^ Despite its ecological significance, the Amazon remains highly vulnerable and is increasingly affected by climate change and anthropogenic pressures.^3,6^

Among these pressures, illegal logging constitutes one of the principal threats to the ecological integrity of the Amazon rainforest. This activity promotes the selective extraction of species with high commercial value, thereby compromising both the structural composition and functional diversity of the ecosystem.^7–9^ Consequently, timber traceability emerges as a central challenge for regulating illegal logging, particularly in remote regions of the Amazon.^10,11^

In Brazil, regulation of the timber production chain is implemented, among other means, through the Document of Forest Origin [*Documento de Origem Florestal* - DOF], an official system that records the transport and storage of native forest products. The DOF is intended to enable the tracking of timber from extraction to its final destination. However, because it relies on manual data entry, the system is vulnerable to fraud, undermining the reliability of the reported information.^8,12^

Brazilian enforcement agencies have adopted complementary techniques to verify the identity and origin of seized timber. Anatomical and DNA-based methods enable taxonomic identification; however, their operational application is constrained by costs, time requirements, and infrastructure limitations, particularly in regional enforcement scenarios within the Amazon.^10,13,14^ Alternatively, stable isotope analysis has emerged as a promising approach, as it reflects environmental conditions during tree growth and allows inferences about geographic origin.^15,16^ Nevertheless, the application of this technique in the Amazon remains restricted due to the scarcity of isotopic databases for commercial species, logistical challenges in accessing remote locations, and insufficient investment in data-collection initiatives and database expansion to support future inspections in the region.

This study presents a database of carbon (δ^13^C) and nitrogen (δ^15^N) isotopic signatures for the widely exploited timber species *Manilkara huberi* across multiple regions of the Amazon. Wood samples were collected in situ, allowing for the construction of a robust reference database to verify the relationship between the geographical origin declared in the DOF and the wood’s isotopic signature. Moreover, the methodology demonstrates operational viability for implementation in regional laboratories in the Amazon, facilitating expeditious analyses to support monitoring and inspection activities within the timber production chain.

The species *Manilkara huberi* (massaranduba) was selected as a model organism owing to its broad distribution in the Amazon, substantial commercial value, and frequent involvement in cases of illegal logging. The working hypothesis is that the isotopic signatures of δ^13^C and δ^15^N exhibit spatial variation among populations, thereby enabling the discrimination of their geographic origins. Accordingly, the objective of this study was to analyze isotopic signatures in individuals of *M. huberi* from distinct regions of the Amazon and to assess their potential as a tool for timber traceability.

## 2 Results

The analysis of nitrogen isotope signatures (δ^15^N) revealed that, in Amazonas, the lowest average value was recorded in Humaitá (−0.12‰), whereas the highest was found in Parintins (1.53‰) (Table 1). Among the populations in this state, the maximum value observed was 2.92‰ (Parintins), and the minimum was −1.86‰ (Humaitá). The greatest standard deviation (±1.04) was identified in the results from Humaitá (Table 1).

**Table 1:** Isotopic Signatures of δ^15^N and δ^13^C in *Manilkara huberi* Wood from Four States in the Amazon^a^

			Nitrogen (δ^15^N)		Carbon (δ^13^C)	
State	Municipality	*N*	Average ± SD	Min-Max	Average ± SD	Min-Max
Amazonas	Humaitá	30	–0.12 ± 1.04	–1.86 | 2.05	–27.83 ± 0.82	–29.67|−26.56
	Itapiranga	20	0.26 ± 0.58	–0.70 | 1.50	–27.15 ± 1.00	–29.50|−26.05
	Parintins	17	1.53 ± 0.74	0.28 | 2.92	–27.76 ± 0.84	–29.26|−25.91
	Silves	20	0.99 ± 0.53	–0.02 | 1.82	–26.99 ± 0.94	–28.99|−25.41
Pará	Aveiro-UPA 1	27	1.02 ± 0.54	–0.12 | 2.32	–26.97 ± 0.48	–27.96|−25.84
	Aveiro-UPA 10	29	3.65 ± 0.50	2.86 | 4.74	–27.11 ± 0.69	–28.64|−26.08
	Belterra-km 117	29	2.46 ± 0.91	0.90 | 4.01	–26.29 ± 0.35	–27.11|−25.63
	Belterra-km 140	28	2.45 ± 0.92	0.71 | 4.06	–26.21 ± 0.56	–27.59|−25.26
	Juruti	20	3.95 ± 0.76	2.85 | 5.37	–27.24 ± 0.61	–28.22|−26.05
Roraima	Caracaraí	29	1.93 ± 0.78	0.09 | 3.44	–27.19 ± 0.65	–28.54|−25.75
	Rorainópolis	30	2.34 ± 0.77	1.02 | 3.57	–27.67 ± 0.92	–29.25|−25.98
Rondônia	Candeias do Jamari	30	4.95 ± 0.73	3.64 | 6.23	–27.35 ± 0.66	–28.44|−26.04
	Itapuã do Oeste	30	2.32 ± 1.25	0.14 | 4.19	–27.28 ± 0.67	–29.04|−26.18

a *N* = number of wood samples analyzed statistically; SD = standard deviation; Min-Max = range of observed values. Values are expressed in δ (‰) relative to the international standards VPDB (δ^13^C) and AIR (δ^15^N).

In Pará, the highest δ^15^N values were observed in Juruti (3.95‰), while the lowest were found in Aveiro-UPA 1 (1.02‰). The maximum recorded value was 5.37‰ (Juruti), and the minimum was −0.12‰ (Aveiro-UPA 1). The greatest standard deviation (±0.92) occurred in Belterra-Km 140 (Table 1).

In Roraima, Rorainópolis exhibited the highest average positive δ^15^N value (2.34‰). Among the populations analyzed in this state, the maximum value recorded was 3.57‰ (Rorainópolis), and the minimum was 0.09‰ (Caracaraí). The greatest standard deviation (±0.78) was observed in Caracaraí (Table 1).

In Rondônia, the highest average δ^15^N value was recorded in Candeias do Jamari (4.95‰). The maximum observed value was 6.23‰ (Candeias do Jamari), and the minimum was 0.14‰ (Itapuã do Oeste). The greatest standard deviation (±1.25) occurred in Itapuã do Oeste (Table 1).

Analysis of carbon isotope ratios (δ^13^C) in Amazonas revealed that the least negative average value was observed in Silves (−26.99‰), while the most negative was observed in Humaitá (−27.83‰) (Table 1). Among the municipalities in Amazonas, the maximum δ^13^C value was −25.41‰ (Silves), and the minimum was −29.67‰ (Humaitá). The greatest standard deviation (±1.00) was observed in Itapiranga (Table 1).

In Pará, the least negative δ^13^C value was observed in Belterra-km 140 (−26.21‰), while the most negative was found in Juruti (−27.24‰). Among the municipalities, the maximum recorded value was −25.26‰ (Belterra-km 140), and the minimum was – 28.64‰ (Aveiro-UPA 10). The greatest standard deviation (±0.69) occurred in Aveiro-UPA 10 (Table 1).

In Roraima, Caracaraí exhibited the least negative mean δ^13^C value (−27.19‰). Among the populations analyzed in this state, the maximum recorded value was −25.75‰ (Caracaraí), and the minimum was −29.25‰ (Rorainópolis). The greatest standard deviation (±0.92) was observed in Rorainópolis (Table 1).

In Rondônia, the least negative average δ^13^C value was observed in Itapuã do Oeste (−27.28‰). Among the municipalities, the maximum recorded value was −26.04‰ (Candeias do Jamari), and the minimum was −29.04‰ (Itapuã do Oeste). The greatest standard deviation (±0.67) occurred in Itapuã do Oeste (Table 1).

Considering the individual wood-sample values of the isotopic signatures and the greatest data dispersion as indicated by standard deviation, the highest dispersions for δ^15^N were observed in Itapuã do Oeste (RO) and Humaitá (AM). For δ^13^C, the highest dispersions occurred in Itapiranga (AM), Silves (AM), and Rorainópolis (RR) (Figures 1 and 2).

**Figure 1:**
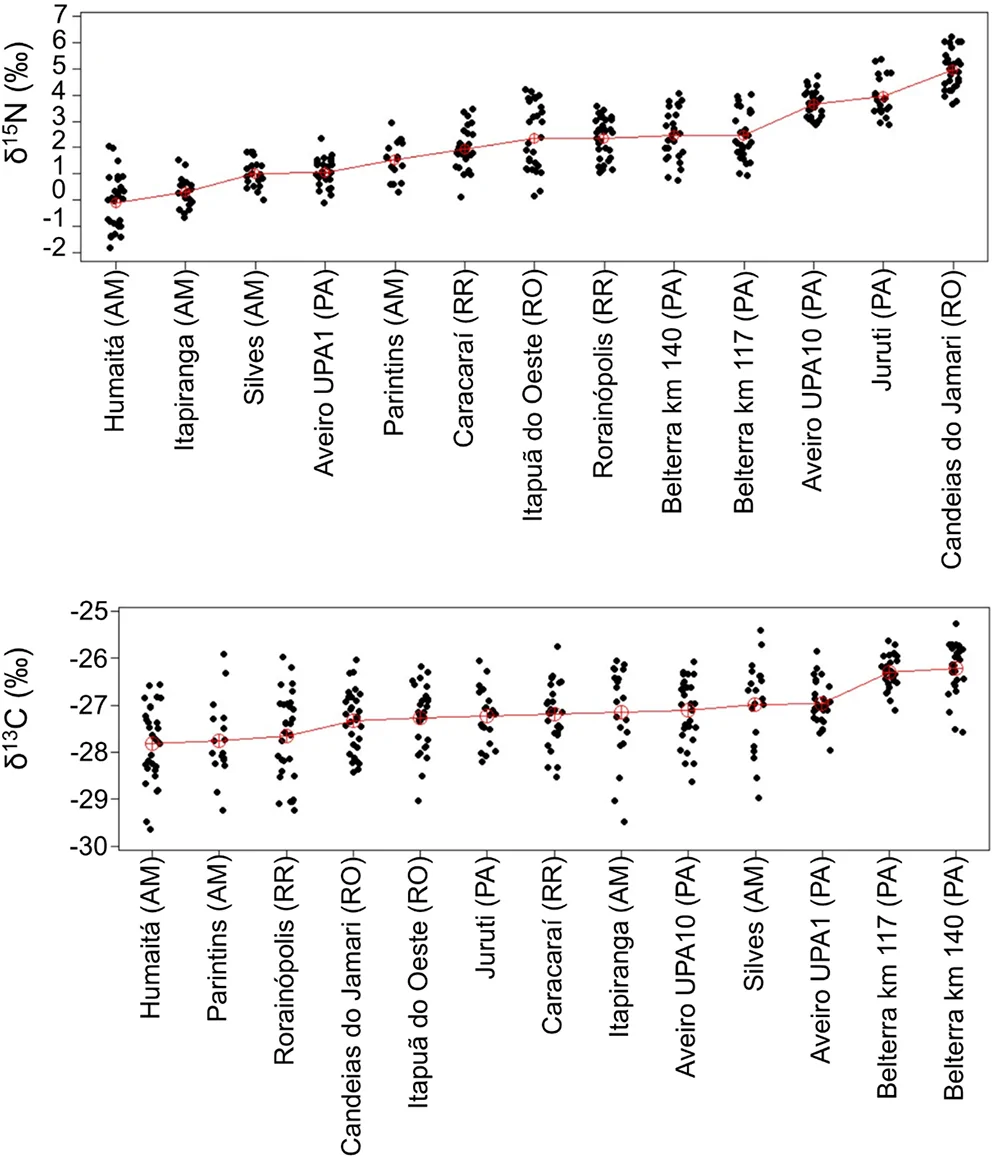
Isotopic signatures of δ^15^N and δ^13^C in wood samples from *Manilkara huberi* in relation to the population average in the Legal Amazon.

**Figure 2:**
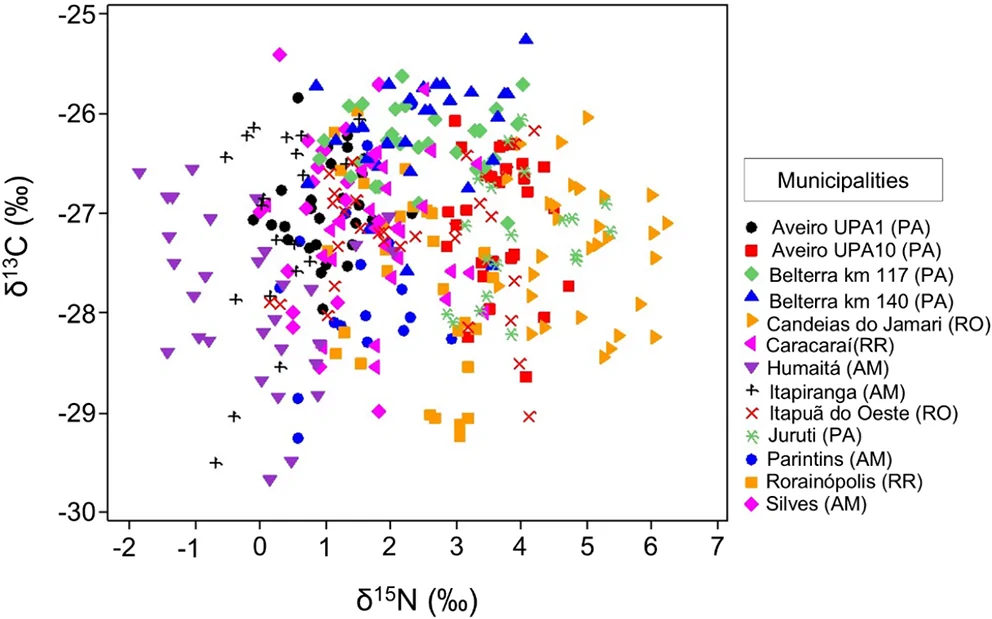
Isotopic signatures of δ^13^C and δ^15^N in wood samples from *Manilkara huberi* collected in 11 municipalities in four states of the Legal Amazon.

Among the municipalities sampled in the Amazon states, the most negative δ^15^N values were observed in Humaitá (AM), whereas the most positive values occurred in Candeias do Jamari (RO) (Figure 1). Regarding δ^13^C, Humaitá (AM) exhibited the most negative average values, while Belterra-km 140 (PA) presented the least negative (Figure 1).

Analysis of variance (ANOVA) conducted on the 13 populations revealed significant differences in δ^15^N and δ^13^C stable isotope values (*p* < 0.01) among the evaluated groups (Table 2).

**Table 2:** Analysis of Variance for δ^15^N and δ^13^C Isotopic Signatures in Wood Samples from *Manilkara huberi* from 13 Different Locations in the Legal Amazon^a^

Nitrogen (δ^15^N)					
Source	GL	SQ	QM	F	P
Municipalities	12	666.53	55.54	82.84	0.000
Error	326	218.57	0.67		
Total	338	885.10			
S	0.82	*R* ^2^ = 75.3%	*R* ^2^(adj) = 74.4%		

Carbon (δ^13^C)					
Source	GL	SQ	QM	F	P
Municipalities	12	77.79	6.48	12.66	0.000
Error	326	166.97	0.51		
Total	338	244.75			
S	0.72	*R* ^2^ = 31.8%	*R* ^2^(adj) = 29.3%		

a GL: Degrees of freedom; SS: Sum of squares; MS: Mean square; F: Fisher’s F value; P: Probability.

In the ANOVA for δ^15^N, the *R* ^2^ values indicated that approximately 75% of the total variation was explained by geographical origin, demonstrating a high discriminatory power of geographical origin. In contrast, for δ^13^C, only about 30% of the variation was attributed to sample origin, reflecting a lower discriminatory power for this isotope.

The mean-comparison test revealed six distinct δ^15^N groups (Table 3), with only the municipality of Candeias do Jamari (RO) forming an isolated group. For δ^13^C, three distinct groups were observed, with greater differences among the populations of Belterra (PA), Rorainópolis (RO), Humaitá, and Parintins (AM) (Table 3). The formation of fewer groups supports the conclusion that geographical origin, as indicated by δ^13^C, possesses lower discriminatory power than δ^15^N.

**Table 3:** Comparison of δ^13^C and δ^15^N Averages in *Manilkara huberi* Wood from 13 Collection Sites across 11 Municipalities and 4 States in the Legal Amazon Region^a^

State	Municipality	δ^15^N	δ^13^C
Rondônia	Candeias do Jamari	4.95 [a]	–27.34 [b]
Pará	Juruti	3.95 [b]	–27.24 [b]
Pará	Aveiro-UPA 10	3.65 [b]	–27.11 [b]
Pará	Belterra-km 117	2.46 [c]	–26.29 [a]
Pará	Belterra-km 140	2.45 [c]	–26.21 [a]
Roraima	Rorainópolis	2.34 [c]	–27.67 [c]
Rondônia	Itapuã do Oeste	2.32 [c]	–27.28 [b]
Roraima	Caracaraí	1.94 [d]	–27.19 [b]
Amazonas	Parintins	1.52 [d]	–27.76 [c]
Pará	Aveiro-UPA 1	1.02 [e]	–26.97 [b]
Amazonas	Silves	0.99 [e]	–26.99 [b]
Amazonas	Itapiranga	0.26 [f]	–27.15 [b]
Amazonas	Humaitá	–0.12 [f]	–27.83 [c]
Average		2.19	–27.14
Coefficient of variation (CV%)		37.31	2.64

a Means followed by the same letter in square brackets within each column do not differ statistically, according to the Scott–Knott test at the 5% probability level. Values are expressed in δ (‰) relative to the international standards VPDB (δ^13^C) and AIR (δ^15^N).

The results of the Linear Discriminant Analysis (LDA), which considered all 13 sampled locations simultaneously, indicated a maximum classification accuracy of 67% for a single locality (see S1–Supporting Information). This value corresponds to the municipality of Candeias do Jamari (RO), where 20 of the 30 observations were correctly classified by the analysis. The lowest accuracy rate (5%) was observed in the municipality of Silves (AM), with only one observation correctly classified (S1-Supporting Information). Of the 339 total observations, the analysis correctly classified 102, representing approximately 30% of the sample (S1-Supporting Information).

As a sensitivity analysis, LOTO validation yielded 27.43% correct classifications, compared with 30.09% obtained using LOOCV, corresponding to a reduction of only 2.66 percentage points. Thus, grouping samples at the tree level during cross-validation did not substantially alter the classification performance or the overall interpretation of the results (see S6-Supporting Information).

A separate LDA was subsequently conducted at the state level. In this analysis, the overall average classification accuracy was 58%, with Amazonas exhibiting the highest accuracy (70%) and Roraima the lowest (41%) (Table [Table tbl4A]).

**4A tbl4A:** Results of Linear Discriminant Analysis (LDA) among States, Grouping Municipalities by State, Based on δ^15^N and δ^13^C Values of *Manilkara huberi*

States	Total Wood Samples	Correct Classifications	Accuracy (%)
Amazonas	87	61	70
Pará	133	72	54
Rondônia	60	38	63
Roraima	59	24	41
Overall accuracy	339	195	58

When separate LDA models were conducted within each state, classification accuracy varied among municipalities (Table [Table tbl4B]). In Amazonas, the highest accuracy was observed in Humaitá (67%), and the lowest in Itapiranga (45%). In Pará, the highest percentage of correct classifications occurred in Aveiro-UPA 1 (96%), and the lowest in Belterra-km 117 (14%). In Roraima, Caracaraí exhibited the highest accuracy (66%), while Rorainópolis had the lowest (47%). In Rondônia, Candeias do Jamari had the highest percentage (97%), compared to Itapuã do Oeste (77%). Overall, municipalities in Rondônia achieved the highest rates of correct classification, while those in Pará had proportions below 30% (Table [Table tbl4B]).

**4B tbl4B:** Linear Discriminant Analysis (LDA) among Municipalities Based on δ^15^N and δ^13^C Values of *Manilkara huberi* [Table-fn t4Bfn1]

Amazonas Municipality	Total Wood Samples	Correct Classifications	Accuracy (%)	95% CI (%)
Humaitá	30	20	67	47.2–82.7
Itapiranga	20	9	45	23.1–68.5
Parintins	17	10	59	32.9–81.6
Silves	20	11	55	31.5–76.9
Overall accuracy	87	50	58	46.9–68.9
Pará Municipality				
Juruti	20	11	55	31.5–76.9
Belterra-km 117	29	4	14	3.9–31.7
Belterra-km 140	28	10	36	18.6–55.9
Aveiro-UPA 1	27	26	96	81.0–99.9
Aveiro-UPA 10	29	15	52	32.5–70.6
Overall accuracy	133	66	50	41.3–58.7
Roraima Municipality				
Caracaraí	29	19	66	45.7–82.1
Rorainópolis	30	14	47	28.3–65.7
Overall accuracy	59	33	56	42.4–68.9
Rondônia Municipality				
Itapuã do Oeste	30	23	77	57.7–90.1
Candeias do Jamari	30	29	97	82.8–99.9
Overall accuracy	60	52	87	75.6–93.8

a 95% CI = exact 95% binomial confidence interval (Clopper–Pearson) for cross-validated classification accuracy.

To compare the geological, pedological (see S2-Supporting Information), and vegetation (see S3-Supporting Information) characteristics of the study areas, data were obtained from the Environmental Information Database (EID) of the Brazilian Institute of Geography and Statistics.^32^ In Amazonas, the principal differences among municipalities arise from Humaitá’s location within a province and subprovince characterized by a predominantly Cenozoic–Indiscriminate Cenozoic geological structure, in contrast to the other sites (S2-Supporting Information). Additionally, this municipality is distinguished by Red-Yellow Dystrophic Argisol soils and predominantly Open Alluvial Rainforest vegetation (S3-Supporting Information).

In Pará, the primary distinction among municipalities is the soil texture of Aveiro-UPA 10, which is classified as clayey, whereas the others possess a medium texture (S2-Supporting Information). In Roraima, both municipalities share similar vegetation types but differ in soil type and texture (S3-Supporting Information). In Rorainópolis, the soil is characterized as dystrophic yellow Argisol with a medium to clayey texture and gently undulating relief. In Caracaraí, the predominant soil type is dystrophic Haplic Tb Gleysol, featuring a sandy to medium texture and flat relief (S2-Supporting Information).

In Rondônia, the predominant vegetation type varies among the areas studied (S3-Supporting Information). In Candeias do Jamari, the vegetation is classified as Open Lowland Rainforest, with soils belonging to the Cenozoic–Indiscriminate Cenozoic structural province and subprovince (S2-Supporting Information). In Itapuã do Oeste, the predominant vegetation is Open Submontane Rainforest, and the soils are associated with the Amazon–Rio Negro Juruena province and structural subprovince.

These differences in soil composition among municipalities reflect underlying lithological variation. In Candeias do Jamari, clay, sand, and laterite predominate, whereas Itapuã do Oeste exhibits a broad lithological diversity, including gneiss, rhyolite, and other metamorphic rocks (S2-Supporting Information).

Joint dispersion analysis of the states indicates that Amazonas and Rondônia exhibit greater population separation when considering the δ^13^C and δ^15^N isotopes of *M. huberi* (Figures 3 and 4A).

**Figure 3:**
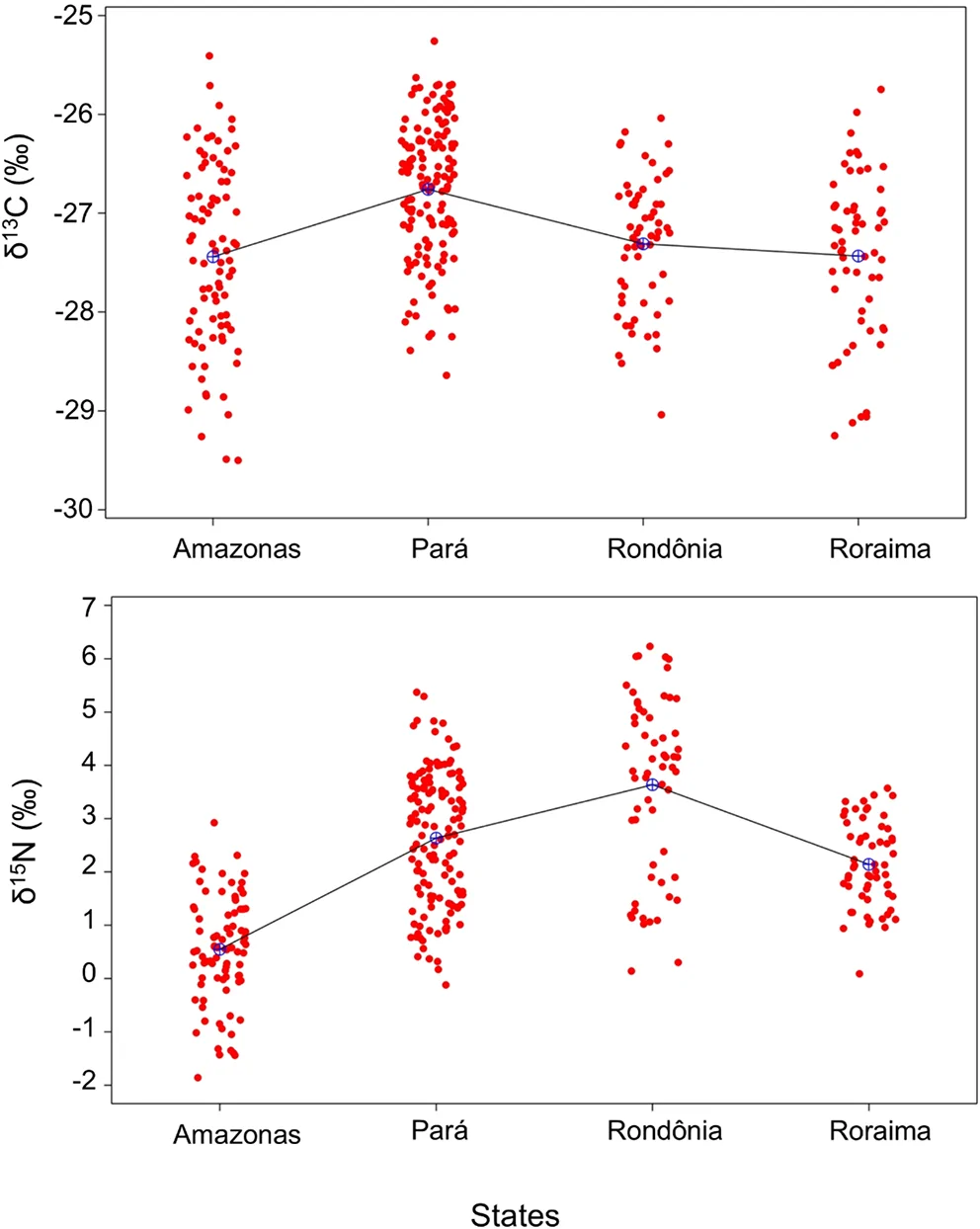
Distribution of δ^13^C and δ^15^N isotopic signatures in *Manilkara huberi* wood samples across states in the Amazon region. Red dots represent individual wood-sample values, and blue circles indicate the mean values for each state.

**Figure 4:**
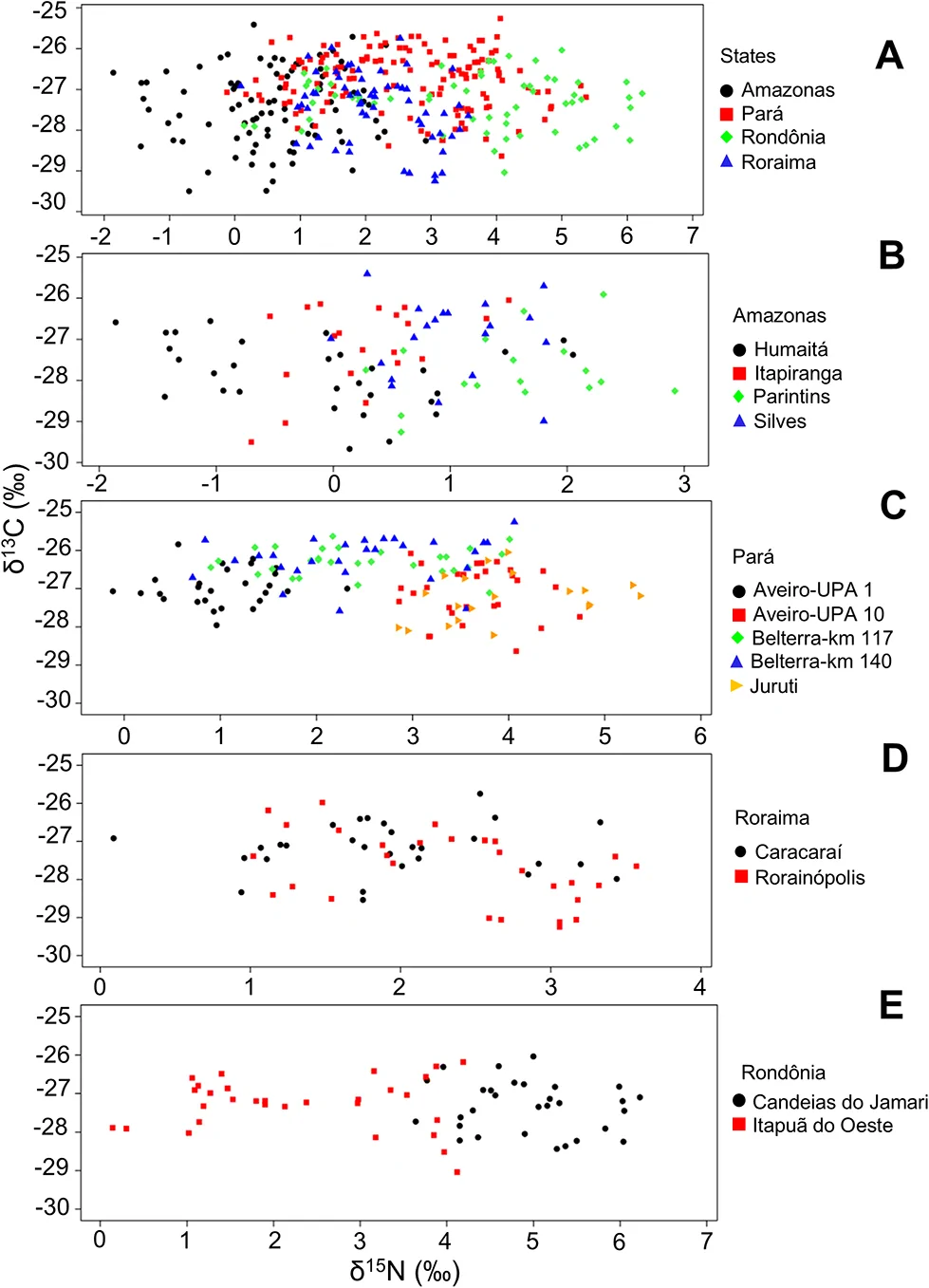
Isotopic signatures of δ^13^C and δ^15^N in *Manilkara huberi* wood samples by state and municipality in the Legal Amazon. (A) States; (B) municipalities in Amazonas; (C) municipalities in Pará; (D) municipalities in Roraima; (E) municipalities in Rondônia.

The separation between the populations of Humaitá and Parintins in Amazonas is considerable (Figure 4B). In Pará, the populations of Aveiro-UPA1 and Juruti can be distinguished with greater precision due to the distance observed between their isotopic signatures (Figure 4C). In Roraima, the isotopic signatures of *M. huberi* populations overlap substantially, impeding clear differentiation among the analyzed areas (Figure 4D). In Rondônia, the separation of sampled areas enables more precise geolocation of populations (Figure 4E).

## 3 Discussion

This study extends the application of stable isotope analyses to natural populations of *Manilkara huberi* distributed across four states within the Amazon region. It highlights the potential of combining carbon (δ^13^C) and nitrogen (δ^15^N) isotopic signatures to distinguish wood origin. The observed differences reflect not only regional environmental variation but also ecophysiological and biogeochemical processes associated with local growing conditions and forest management histories. Consequently, the observed isotopic variability underscores the value of stable isotopes as complementary tools for ecological and forensic traceability. These results also provide essential baseline information for future studies on timber origin verification in the Amazon.

The populations of *M. huberi* exhibit distinct carbon (δ^13^C) and nitrogen (δ^15^N) isotopic signatures, enabling differentiation among municipalities in the Amazon. Although δ^13^C signatures were more similar across populations, they were sufficient to distinguish the groups. δ^13^C serves as an effective indicator of functional variability in tropical trees and demonstrates the importance of the canopy effect on forest isotopic composition.^17–19^ In Amazonas, the observed values indicate denser canopies, which lead to greater isotopic discrimination due to reduced light availability in the understory.^20^ In Pará, more negative δ^13^C values suggest greater water-use efficiency, possibly associated with taller trees and structural variations within the forest.^20^ The largest difference observed between areas was 1.62‰, consistent with patterns described for widely distributed species, in which δ^13^C tends to exhibit relative spatial stability due to strong genetic and physiological control of this trait.^21^

In contrast, nitrogen isotope signatures (δ^15^N) exhibited substantial spatial variation and contributed most strongly to the discrimination among the populations analyzed. δ^15^N primarily reflects nitrogen cycling processes within ecosystems and is influenced by soil nutrient availability, gaseous losses due to denitrification and volatilization, and local edaphic and geological factors. In environments with higher nitrogen availability, plants and soils exhibit isotopic enrichment in δ^15^N due to the preferential loss of ^14^N to the atmosphere.^19^ The differences observed between geographically proximate locations, such as Juruti (PA) and Parintins (AM), corroborate evidence that nitrogen biogeochemical processes can vary at relatively small spatial scales in the Amazon, being strongly modulated by geology, pedology, and vegetation composition.^20^ These findings indicate that spatially structured variation in δ^15^N, arising from local environmental and biogeochemical processes, can contribute substantially to distinguishing the geographic origin of tropical woods.

Most of the samples analyzed were collected from forest concession areas, environments subject to disturbance from selective tree extraction, despite being classified as low-impact. Exceptions included Humaitá (AM), characterized as an igapó area with minimal anthropogenic interference, and Juruti (PA), a terra firme area subject to greater human intervention. In this context, the observed isotopic differences reflect not only regional climatic variation but also the degree of anthropogenic influence and the history of forest management in the areas studied. Previous research demonstrates that disturbances such as deforestation and forest fires increase nitrogen mobility in the soil, promoting isotopic enrichment in δ^15^N due to the preferential loss of ^14^N.^22^

When all sampled areas were analyzed collectively, linear discriminant analysis (LDA) showed reduced ability to accurately classify sample origins, suggesting that including multiple populations may diminish the discriminatory power of the selected predictors. The observed decrease in accuracy (30%) aligns with established limitations of LDA in ecological contexts characterized by substantial environmental heterogeneity and multivariate overlap among groups. LDA assumes both homogeneity of covariance matrices and linear separability between classes-assumptions that are seldom fully satisfied at broad geographic scales, such as in the Amazon, where continuous environmental gradients and complex ecophysiological interactions commonly result in partially overlapping predictor distributions.^23,24^ Additionally, research on isotopic traceability indicates that classification accuracy typically declines as the number of groups and intraspecific variability increase, particularly when predictors are influenced by multiple, correlated environmental factors.^25,26^ In this context, the observed reduction does not imply the absence of a geographic signal; rather, it reflects the limitations of linear approaches in capturing complex patterns at large spatial scales.

These findings indicate that analyses performed at smaller spatial scales, such as by municipality or within individual states, or that include additional environmental variables, tend to improve LDA efficiency. Notably, the observed classification accuracy of approximately 58% at the state level supports the premise that the discriminatory power of isotopic markers is scale-dependent, with higher performance when environmental variability is more constrained and ecological gradients are more homogeneous. This trend is consistently documented in the literature, where discriminant models yield superior performance at regional scales or when applied to environmentally coherent subsets, thereby reducing class overlap.^26,27^ Within this context, nitrogen emerged as a particularly robust predictor of location within the same state, potentially reflecting variation in soil properties, nitrogen cycling, vegetation composition, and disturbance history. Carbon, meanwhile, served as a complementary variable, reflecting environmental conditions during photosynthesis and thus contributing to the discrimination of wood from different populations of the same species.^27–30^

The results obtained directly address the need for independent methods to validate information presented in DOF. The environmentally structured isotopic variability observed in this study, grounded in well-established ecological processes, supports the potential use of isotope analysis as a complementary tool for evaluating the consistency of declared wood origins, particularly in the Amazon. Consequently, stable isotopes may provide additional scientific information for assessing declared provenance alongside existing documentation.

According to data from the Brazilian Institute of Environment and Renewable Natural Resources,^31^ *M. huberi* is the most exploited among the 25 principal native timber species in Brazil. In the Amazon, merely 2% of the available timber species account for more than half of the authorized exploitation between 2007 and 2020, with *M. huberi* representing the highest volume: 11,181,124.7 m^3^. This is followed by *Erisma uncinatum* (cedrinho), *Goupia glabra* (cupiúba), *Dinizia excelsa* (angelim vermelho), and *Hymenaea courbaril* (jatobá).^32^ To curb the illegal extraction of these species, frequently targeted by international markets, particularly China, the USA, Japan, and countries of the European Union (Germany, Italy, The Netherlands, and Belgium), as well as the United Kingdom,^33^ the Brazilian government, through the Federal Police, has intensified operations to combat such crimes within the national territory.

Environmental crimes, such as illegal deforestation in primary and secondary Amazonian forests, are an increasing cause for concern.^3,6^ In light of the impacts of global warming, land-use sectors have been actively discussing strategies to reconcile the sustainable use of forest resources with the Sustainable Development Goals (SDGs) and ensure the well-being of future generations.^1,34,35^ Among the most promising strategies for combating the illegal timber trade are strengthening public security agencies and establishing international alliances dedicated to disrupting the illegal Amazonian timber supply chain.^36–38^

The georeferenced isotopic database developed for *M. huberi* constitutes an important reference for environmental monitoring and verification of the origin of its wood. The results demonstrate the potential of stable isotope analysis to characterize spatially structured variation in the isotopic signatures of this commercially valuable Amazonian species, thereby expanding the application of established isotopic methodologies for forest traceability in the Amazon. This study contributes to strengthening wood traceability systems by providing critical information for the monitoring and prevention of illegal logging. Since wood is not isotopically homogeneous, preserving the variability observed among different sampling points within the same tree offers a more realistic representation of operational traceability scenarios, in which forensic samples may originate from different portions of logs, boards, or processed timber products. Previous studies have also documented within-tree isotopic variability and its relevance for geographic traceability applications.^17,18^

The inclusion of additional isotopes, such as oxygen and hydrogen, enhances the accuracy of isotopic traceability for wood from native Amazonian species.^17,28,39^ The integration of multiple elemental signatures can improve accuracy rates in determining wood origin using LDA, potentially providing additional scientific support for the evaluation of information contained in DOF and for future initiatives aimed at combating illegal trade. Incorporating this tool into official certification systems, such as IBAMA (Instituto Brasileiro do Meio Ambiente e dos Recursos Naturais Renováveis), FSC (Forest Stewardship Council), and CITES (Convention on International Trade in Endangered Species of Wild Fauna and Flora), could contribute to the further development of complementary approaches for tropical wood traceability. Future research should include external validation, the use of an independent test set, and the verification of commercial timber.

## 4 Conclusions

Combined analysis of carbon (δ^13^C) and nitrogen (δ^15^N) isotopes demonstrated potential for the geographic discrimination of populations of the same species. Performance varied according to spatial scale and geographic context. The resulting isotopic database can be used to compare samples obtained during enforcement actions involving the species.

The observed differences in δ^13^C and δ^15^N isotopic ratios among individuals of the same species provide a basis for geographic traceability. This is particularly relevant in regions with pronounced contrasts in geology, vegetation, and climate, which may enhance the discriminatory power of the method. Classification accuracy varied according to spatial scale. Higher performance was observed in municipality-level comparisons than in analyses conducted across states. Therefore, the higher classification accuracies observed for specific municipalities should not be interpreted as representative of overall geographic traceability performance.

This finding underscores the need to incorporate additional predictor isotopes, such as δ^2^H and δ^18^O, as well as complementary methodologies, to improve traceability accuracy. These results support the potential use of stable isotopes as complementary scientific and forensic tools. They may support timber origin verification, monitoring of logging activities, and environmental control measures in the Amazon. The current isotopic database should therefore be regarded as a reference framework, and its operational forensic application requires further validation using independent commercial or seized timber samples.

## 5 Materials and Methods

### 5.1 Origin and Sampling of the Material

This study examines wood sold under the commercial name “massaranduba” derived from the botanical species *Manilkara huberi*, a species widely exploited and used in the timber industry. The trees were identified in the field through botanical analyses conducted by parabotanists and forestry engineers, based on morphological characteristics, including leaves, flowers, fruits, and trunks, as well as general organoleptic characteristics, with special attention to the abundance and consistency of the latex characteristic of this species.

For this research, four states in the Amazon region were selected: Pará, Amazonas, Roraima, and Rondônia, all of which exhibit the highest accumulated deforestation rates in the Legal Amazon.^40^ Within each state, areas primarily managed under forest concessions were chosen for wood sample collection. In Pará, the selected municipalities were Juruti (1), Aveiro (2), and Belterra (2). In Amazonas, sampling took place in Humaitá (1), Parintins (1), Itapiranga (1), and Silves (1). In Roraima, wood samples were collected in Rorainópolis (1) and Caracaraí (1). In Rondônia, the selected sites were Candeias do Jamari (1) and Itapuã do Oeste (1) (Figures 5 and 6). Data on meteorological variables, geological and soil characteristics, and forest types for each area are presented in Tables 5.

**Figure 5:**
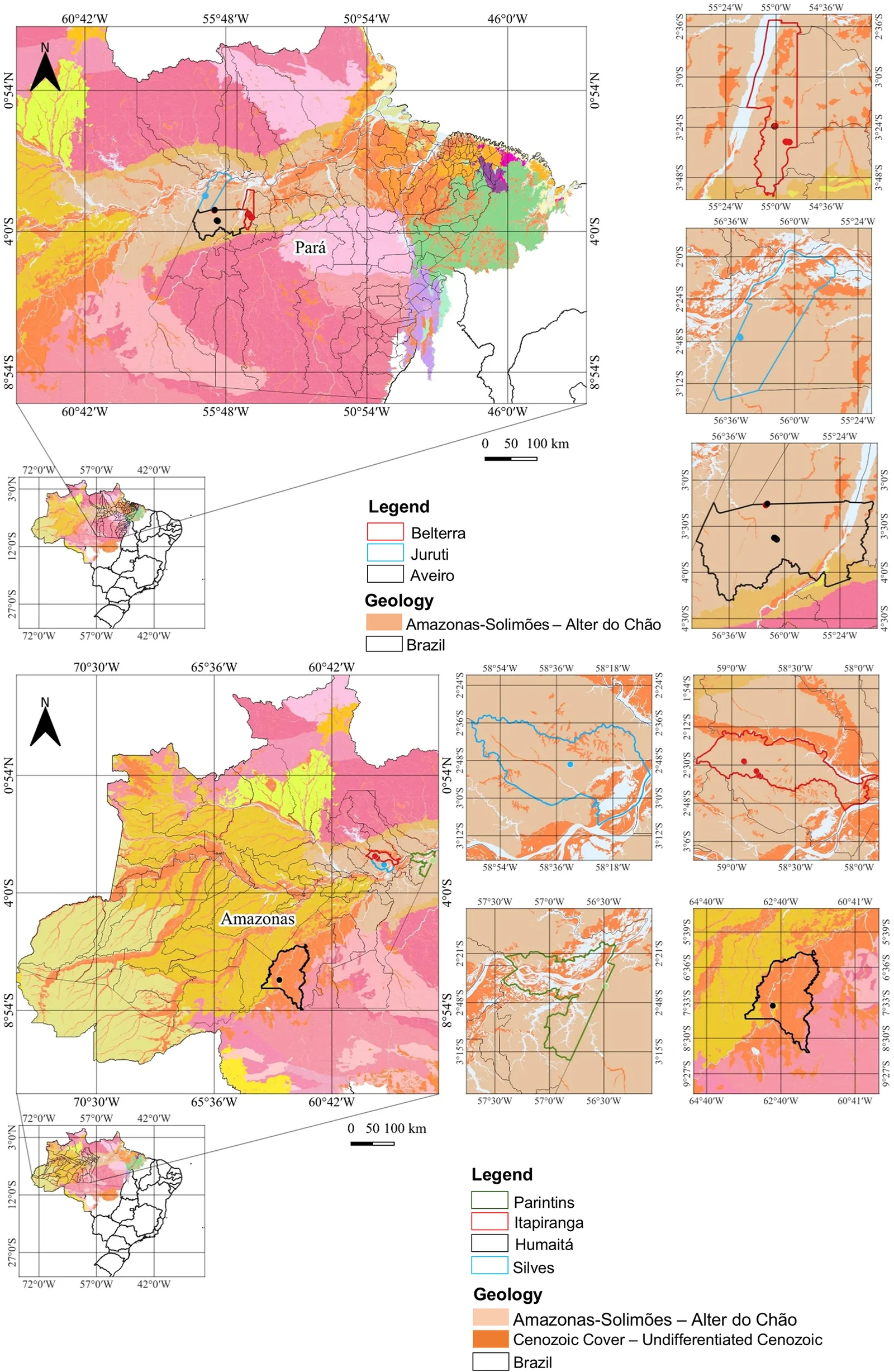
Geographic distribution of *Manilkara huberi* trees in the states of Pará and Amazonas. Source: Prepared by the author in QGIS using IBGE (2021) territorial boundaries; SIRGAS 2000.

**Figure 6:**
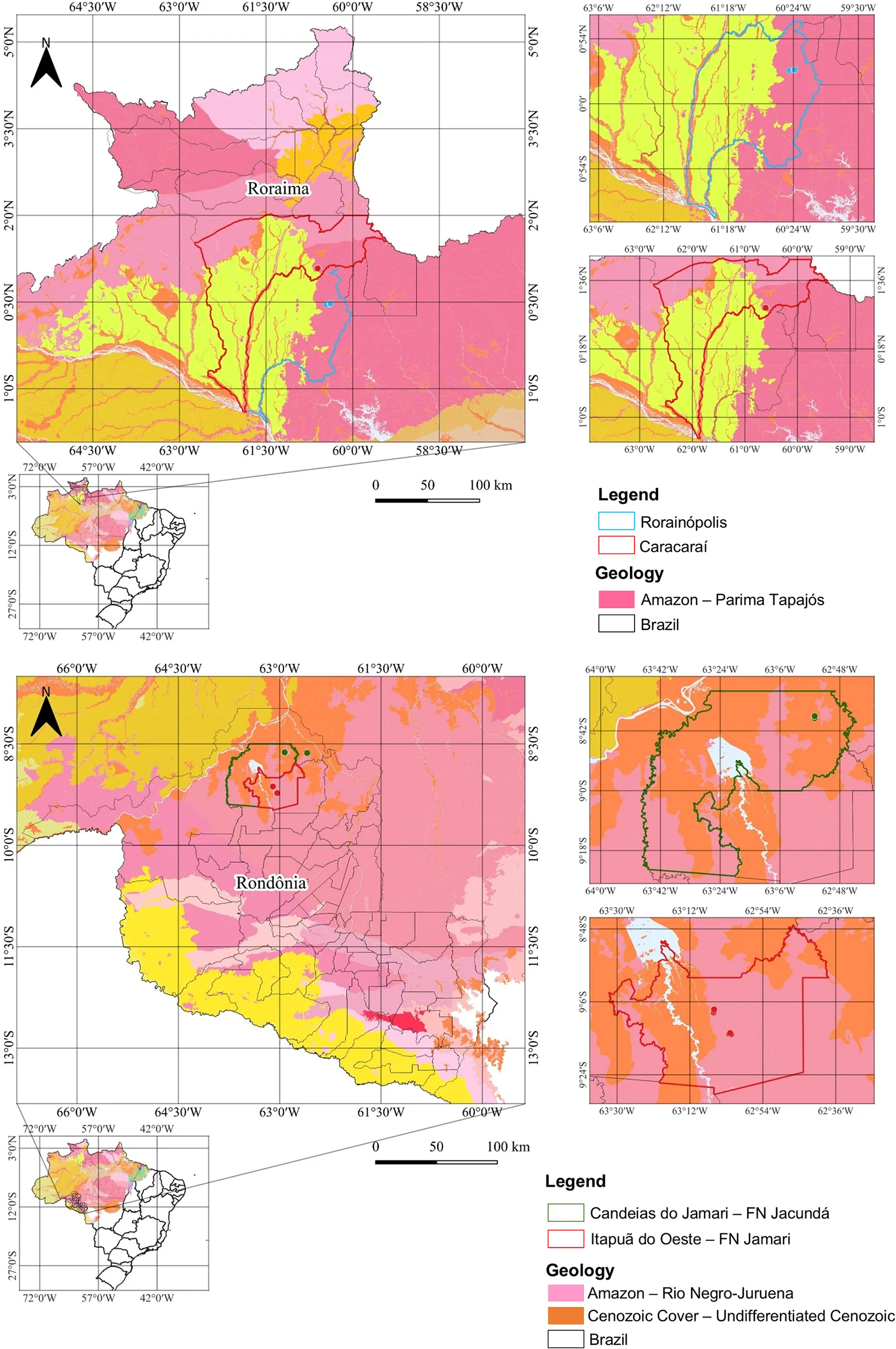
Geographic distribution of *Manilkara huberi* trees in the states of Roraima and Rondônia. Source: Prepared by the author in QGIS using IBGE (2021) territorial boundaries; SIRGAS 2000.

**Table 5:** Climatic Characteristics of the Sampling Areas during the Study Period (07 December 2022 to 21 August 2023)^a^,^b^

State	Sampling municipality	INMET weather station	Station code	Precip. (mm)	Mean Temp. (°C)	Max Temp. (°C)	Min Temp. (°C)	RH (%)
Pará	Juruti	Belterra	82246	1565.4	32	34	30	92
Pará	Aveiro	Belterra	82246	1565.4	32	34	30	92
Pará	Belterra	Belterra	82246	1565.4	32	34	30	92
Amazonas	Humaitá	Humaitá	A112	208.4	27	35	30	79
Amazonas	Parintins	Parintins	A123	1272.4	27	35	30	79
Amazonas	Silves	Itacoatiara	82336	1703.7	32	35	30	79
Amazonas	Itapiranga	Itacoatiara	82336	1703.7	32	35	30	79
Roraima	Caracaraí	Caracaraí	82042	800.0	24	25	23	83
Roraima	Rorainópolis	Boa Vista	82024	2646.9	30	35	24	76
Rondônia	Candeias do Jamari	Porto Velho	A925	725.4	27	28	26	^a^
Rondônia	Itapuã do Oeste	Porto Velho	A925	725.4	27	28	26	^a^

a Climatic data were obtained from the nearest INMET automatic weather station available for each sampling area. Identical values among municipalities may occur when the same weather station was referenced. RH = relative humidity; Precip. = precipitation.

b No data available for the specified period. Source: INMET Meteorological Database (https://bdmep.inmet.gov.br/).

Ten individual trees were sampled at each location between August 2022 and July 2023, resulting in a total of 130 individual trees. The minimum distance between sampled trees ranged from 80 to 100 m. Trees selected for sampling had a diameter at breast height (DBH) of at least 50 cm. All trees were georeferenced to facilitate the construction of geolocation maps and the validation of official databases for tracing species origin. Latitude and longitude were recorded during fieldwork in degrees, minutes, and seconds using a portable GPS device.

### 5.2 Wood Sampling Procedure

For each tree, two to three wood-sampling holes were drilled at 1.30 m above the base of the tree, corresponding to the diameter at breast height (DBH). The number of wood samples was determined by field accessibility and logistical constraints encountered during expeditions in remote Amazonian forests. Whenever possible, three wood samples were collected per tree. However, in some locations (Silves, Itapiranga, Parintins, and Juruti), only two wood samples could be obtained per tree.

At each designated sampling point on the trunk, the surface wood was cleaned by scraping with a machete to remove impurities adhering to the bark or cambium. Wood samples were collected using a battery-powered drill (model: Makita 40 V Li-Ion 140 N m) fitted with 1/4 in. drill bits. The initial 5 to 7 cm of drill shavings from each hole were discarded to ensure that only sapwood and heartwood were sampled. This process was repeated until 5–10 g of wood shavings per hole were obtained. Samples were placed in paper bags and labeled with a matrix number and collection site.

All sampling wounds were sealed with silicone gel to prevent microorganism proliferation. Wood samples were stored in the field in custom-built ovens maintained at 40–50 °C to preserve their integrity until the end of the expeditions (3–4 days). Subsequently, the material was transported to the Forensic Laboratory of Stable Isotopes at the Federal Police Superintendence of Amazonas (SR/PF/AM) for preparation and analysis.

### 5.3 Wood Sample Preparation and Analysis Protocol

After field collection, wood samples were dried in an oven at 40 °C for up to 1 day prior to sample preparation. The material was then transferred to 50 cm^3^ grinding jars containing 25 mm diameter AISI 304 stainless steel spheres and pulverized using a benchtop vibratory mill (MM 400-Retsch) for 2 min at 30 s^–1^. Following pulverization, the material was dried for 2 h at 60 °C. The resulting powder was then transferred to 2 mL microtubes. Each tube was labeled with the tree identification number, sample number, and collection site, and stored in a desiccator until chemical analyses commenced.

To determine the carbon and nitrogen isotopic ratios in wood, a hierarchical sampling and analytical protocol was implemented. For each tree, two or three separate sampling holes were drilled into the trunk. One wood sample was collected from each hole. The total number of samples per tree depended on field accessibility and logistical constraints encountered during the collection of biological material. Each sample was analyzed individually using isotope ratio mass spectrometry, with three consecutive readings performed for each sample as technical replicates to validate analytical precision. These repeated readings were conducted solely to verify the precision of the isotopic determinations. For all samples, the mean value from the three readings remained within the laboratory’s established precision limits (maximum acceptable standard deviation in triplicate: 0.40‰ for δ^15^N and 0.15‰ for δ^13^C; unpublished data). After the validation of analytical quality, the average of the three readings was used to represent the wood sample from each collection hole.

The two or three wood samples obtained from each tree (collected at different drilling points) were treated as distinct wood samples and recorded as separate observations in the data set, without being considered independent biological replicates. This approach was chosen because wood is an inherently heterogeneous biological material. Furthermore, isotopic composition can vary among different portions of the same trunk due to anatomical, physiological, and microenvironmental factors.^17,18^ Removing material from different drilling locations enabled the characterization of within-tree isotopic variability, which is important for forensic timber traceability applications, where samples may originate from various portions of commercial wood products. Therefore, statistical analyses (ANOVA and LDA) were performed at the wood-sample level, while recognizing that samples obtained from the same tree belong to the same biological unit and are not statistically independent. For each determination, 4.97–5.08 mg of wood powder was weighed into tin capsules (5.0 mm × 8.0 mm) using an analytical balance with a resolution of 0.01 mg.

Encapsulated organic samples were analyzed with a Flash EA IsoLink CN elemental analyzer (Thermo Fisher Scientific, Bremen, Germany) coupled to a Delta V Advantage isotope ratio mass spectrometer (Thermo Fisher Scientific, Bremen, Germany) via the ConFlo IV interface. Combustion of samples occurred in an oxidation reactor at 1020 °C, followed by reduction on copper at 650 °C.

The generated gases were carried by a continuous helium (He 5.0) flow and separated using a chromatographic column coupled to a ramped GC oven (Bremen, Germany). The heating program began at 70 °C, was maintained for 250 s, then increased to 240 °C and held for 500 s (see S4-Supporting Information). This temperature ramp was essential to ensure effective gas separation and complete elution of SO_2_ produced during combustion.

Following chromatographic separation, the gases were introduced into a mass spectrometer via the ConFlo IV interface, where they were ionized and analyzed for isotopic ratios. Data acquisition and processing were conducted using Isodat 3.0.0.83 software (Service Pack 3.0.94.12; Thermo Foundation 2.0) (see S5-Supporting Information).

Calibration of the CO_2_ and N_2_ cylinders was conducted using international reference materials, specifically the IAEA-602 (δ^13^C = −28.85 ± 0.04‰) and IAEA-N-2 (δ^15^N = 20.41 ± 0.12‰) standards.

Isotopic ratios were expressed in delta notation (δ), relative to the international standards Vienna Pee Dee Belemnite (VPDB) for carbon and atmospheric air (AIR) for nitrogen, as defined by the following equation

$$\delta =\left(\frac{R\_sample}{R\_s\tan dard}-1\right)1000$$

Where: *R*_sample is the ^13^C:^12^C or ^15^N:^14^N ratio of the sample; *R*_standard is the ^13^C:^12^C or ^15^N:^14^N ratio of the corresponding reference gas. The result is multiplied by 1000, yielding the delta (δ) value in per mil (‰). For these calculations, reference values (*R*_standard) of 0.0112021 for VPDB (^13^C:^12^C) and 0.0036433 for AIR (^15^N:^14^N) were used.^41^

For analytical quality control, a reference material of *Handroanthus serratifolius* (Vahl) S.O.Grose (yellow ipê, code “CTR Ipê”, wood), previously calibrated at the Forensic Stable Isotope Laboratory of the Federal Police Superintendence of Amazonas (SR/PF/AM) with δ^13^C values of −26.96 ± 0.05‰ and δ^15^N values of −6.90 ± 0.30‰ (±2σ control limits), was employed as an internal standard. This standard was analyzed in quadruplicate at the beginning and end of each analytical sequence, as well as for every set of approximately 78 samples, thereby enabling continuous monitoring of instrumental stability and measurement accuracy.

### 5.4 Data Processing

Prior to statistical analysis, the data set was examined for extreme observations using boxplot analysis based on the interquartile range (IQR) criterion. Observations falling outside the thresholds defined by Q1 – 1.5 × IQR and Q3 + 1.5 × IQR were classified as potential outliers and excluded from further analysis.

After quality control and exclusion of the 11 observations identified as statistical outliers, the final data set comprised 339 wood-sample observations. This final data set was consistently used for the ANOVA and LDA analyses and for the corresponding tables, figures, and Supporting Information, unless otherwise explicitly indicated.

Descriptive statistics (mean, minimum, maximum, and standard deviation) were calculated for the δ^15^N and δ^13^C values of the collected wood samples. Differences in mean isotopic values among sampling locations were evaluated using the Scott-Knott test,^42^ implemented in the Genes software version 21.^43^ This test was selected because it groups means into statistically distinct clusters, thereby facilitating the interpretation of differentiation patterns among sampling locations.

Multivariate Linear Discriminant Analysis (LDA) was conducted to assess the differentiation between groups of wood samples and evaluate classification performance by region of origin. Model performance was evaluated using leave-one-out cross-validation (LOOCV), in which each observation was successively omitted from the training set and subsequently classified using discriminant functions fitted to the remaining observations. This procedure was applied to estimate the model’s predictive ability and minimize biases associated with classification evaluation.

Additionally, leave-one-tree-out (LOTO) cross-validation was performed as a sensitivity analysis, in which all wood samples originating from the same tree were jointly excluded from the training data set at each iteration, thereby accounting for the hierarchical sampling structure (see S6–Supporting Information).

LDA and cross-validation analyses were performed using Minitab Statistical Software version 22.5 (Minitab LLC, State College, PA, USA).

The evaluation metrics comprised: (i) overall classification accuracy obtained by cross-validation; (ii) group-wise sensitivity, representing the proportion of correctly classified wood samples within each class; (iii) the confusion matrix, which details classification patterns and misclassifications among groups; and (iv) distances between groups in the discriminant space, facilitating assessment of the degree of separation between classes.

Analyses were performed using both the complete data set and a stratified approach, with LDA applied separately for each state at two scales of analysis. Separate scatter plots and data sets were generated for each area to visualize isotopic signature patterns within each locality.

## Supplementary Material



## Notes

Data include detailed results of linear discriminant analysis (LDA), soil characteristics, vegetation classification, images from the heating program, and chromatograms of carbon and nitrogen isotopes obtained using an EA–IRMS system (PDF)
