# Supplementary material for: Traceability of Manilkara huberi Using Stable Isotope Ratio Analysis to Support Deforestation Prevention in the Amazon
Source: ACS Omega. 2026 Sep 22;11(39):58773–84. doi: 10.1021/acsomega.6c03050 (PMC13647964; doi:10.1021/acsomega.6c03050)
Supplement: Supplementary file 1 [file ao6c03050_si_001.pdf]

SUPPORTING INFORMATION

Traceability of *Manilkara huberi* Using Stable Isotope Ratio Analysis to Support Deforestation Prevention in the Amazon

Helinara L. V. Capucho[1], José H. de Oliveira[2], Ricardo Lopes[3], Santiago L. F. Ramos[1], Ananda V. de Aguiar[4], Carlos H. S. G. Meneses[5], Ricardo L. Marques[2], Fabio de C. Borba[2], Gabriela B. Nardoto[6], Maria T. G. Lopes[1]*

[1] Faculty of Agricultural Sciences, Federal University of Amazonas (UFAM), Manaus, AM, Brazil.

[2] Technical-Scientific Sector, Federal Police (Brazil), Manaus, AM, Brazil.

[3] Brazilian Agricultural Research Corporation (Embrapa), Western Amazon Agroforestry Research Center, Manaus, AM, Brazil.

[4] Brazilian Agricultural Research Corporation, Embrapa Floresta, Colombo, PR, Brazil.

[5] Department of Biology, State University of Paraíba (UEPB), Campina Grande, PB, Brazil.

[6] Institute of Biological Sciences, University of Brasília (UnB), Brasília, DF, Brazil.

*Corresponding author: mtglopes@ufam.edu.br

Table S1. Joint classification of groups and percentage of correct classifications generated by Linear Discriminant Analysis (LDA) using stable carbon ($\delta^{13}C$) and nitrogen ($\delta^{15}N$) isotopes as predictors of the origin locality for wood samples of *Manilkara huberi*, with classification performance evaluated by cross-validation.

Response Variable for Linear Discriminant Analysis	Sites												
**Predictor Variables**	**$\delta^{15}N$; $\delta^{13}C$**												
Classification Groups	Aveiro UPA 10 (PA)	Aveiro UPA 1 (PA)	Belterra Km 117 (PA)	Belterra Km 140 (PA)	Candeias do Jamari (RO)	Caracaraí (RR)	Humaitá (AM)	Itapiranga (AM)	Itapuã do Oeste (RO)	Juruti (PA)	Parintins (AM)	Rorainópolis (RR)	Silves (AM)
Sample Size	29	27	29	28	30	29	30	20	30	20	17	30	20
	Actual Classification Groups												
Within-Group Analysis	Aveiro UPA	Aveiro	Belterra	Belterra Km	Candeias	Caracaraí	Humaitá	Itapiranga	Itapuã do	Juruti (PA)	Parintins	Rorainópolis	Silves

	10 (PA)	UPA 1 (PA)	Km 117 (PA)	140 (PA)	do Jamari (RO)	(RR)	(AM)	(AM)	Oest e (RO)		(AM)	(RR)	(AM)
Aveiro UPA 10 (PA)	13	0	6	4	2	2	0	0	6	10	0	1	0
Aveiro UPA 1 (PA)	0	6	4	3	0	3	0	2	6	0	1	2	9
Belterra Km 117 (PA)	1	0	3	9	0	4	0	0	1	0	1	1	1
Belterra Km 140(PA)	1	0	13	9	0	1	0	1	0	0	1	1	1
Candeias do Jamari (RO)	2	0	0	0	20	0	0	0	0	6	0	0	0
Caracaraí (RR)	0	2	2	1	0	5	3	0	4	0	1	4	1
Humaitá (AM)	0	0	0	0	0	0	16	5	2	0	3	0	2
Itapiranga (AM)	0	5	0	0	0	1	7	8	0	0	1	0	2
Itapuã do Oeste (RO)	2	1	1	0	0	3	0	0	3	0	0	5	0
Juruti (PA)	8	0	0	0	8	1	0	0	4	2	0	1	0
Parintins (AM)	0	5	0	0	0	4	4	0	2	0	5	3	3
Rorainópolis (RR)	2	0	0	1	0	3	0	0	1	2	4	11	0
Silves (AM)	0	8	0	1	0	2	0	4	1	0	0	1	1
Total Samples	29	27	29	28	30	29	30	20	30	20	17	30	20
Number of Correct Classifications	13	6	3	9	20	5	16	8	3	2	5	11	1
**Percentage of Correct Classifications (%)**	45	22	10	32	67	17	53	40	10	10	29	37	5

**Total Number of Observations**	339
**Number of Correct Observations**	102
**Classification Certainty Percentage (%)**	30

Table S2. Geological and pedological characteristics of municipalities where *Manilkara huberi* samples were collected.

Pará

Municipality	Juruti	Aveiro<br>UPA 1	Aveiro<br>UPA 10	Belterra<br>km 140	Belterra<br>km 117
Dominant Structural Province	Amazonas-Solimões	Amazonas-Solimões	Amazonas-Solimões	Amazonas-Solimões	Amazonas-Solimões
Dominant Structural Subprovince	Alter do Chão	Alter do Chão	Alter do Chão	Alter do Chão	Alter do Chão
Geological Era of Formation	Phanerozoic Era, Mesozoic Period, Early Cretaceous Epoch, Albian Age	Phanerozoic Era, Mesozoic Period, Early Cretaceous Epoch, Albian Age	Phanerozoic Era, Mesozoic Period, Early Cretaceous Epoch, Albian Age	Phanerozoic Era, Mesozoic Period, Early Cretaceous Epoch, Albian Age	Phanerozoic Era, Mesozoic Period, Early Cretaceous Epoch, Albian Age
Dominant Lithologies	Sandstone, siltstone, claystone, conglomerate, and laterite.	Sandstone, siltstone, claystone, conglomerate, and laterite.	Sandstone, siltstone, claystone, conglomerate, and laterite.	Sandstone, siltstone, claystone, conglomerate, and laterite.	Sandstone, siltstone, claystone, conglomerate, and laterite.
Soil Type	LAd - Latossolo Amarelo Distrófico	LAd - Latossolo Amarelo Distrófico	LAd - Latossolo Amarelo Distrófico	LAd - Latossolo Amarelo Distrófico	LAd - Latossolo Amarelo Distrófico
Texture	medium	medium	clayey	medium	medium
Relief Type	Flat to gently undulating	Gently undulating to undulating	Gently undulating	Gently undulating	Gently undulating
Amazonas					
Municipality	Humaitá	Itapiranga	Parintins	Silves	
	Cenozoic Sedimentary Cover	Amazonas-Solimões	Amazonas-Solimões	Amazonas-Solimões	

Dominant Structural Province				
Dominant Structural Subprovince	Undifferentiated Cenozoic Sedimentary Cover	Alter do Chão	Alter do Chão	Alter do Chão
Geological Era of Formation	Phanerozoic Eon, Cenozoic Era, Quaternary Period, Holocene Epoch	Phanerozoic Eon, Mesozoic Era, Early Cretaceous Epoch, Albian Age	Phanerozoic Eon, Mesozoic Era, Early Cretaceous Epoch, Albian Age	Phanerozoic Eon, Mesozoic Era, Early Cretaceous Epoch, Albian Age
Dominant Lithologies	Silty sand, clay, conglomerate, peat, gravel, and siltstone.	Sandstone, siltstone, claystone, conglomerate, and laterite.	Sandstone, siltstone, claystone, conglomerate, and laterite.	Sandstone, siltstone, claystone, conglomerate, and laterite.
Soil Type	Pvad - Argissolo Vermelho-Amarelo Distrófico	LAd - Latossolo Amarelo Distrófico	LAd - Latossolo Amarelo Distrófico	LAd - Latossolo Amarelo Distrófico
Texture	Medium to clayey texture	Very clayey texture	Medium texture	Very clayey to clayey texture
Relief Type	Flat relief	Flat to gently undulating relief	Gently undulating relief	Flat to gently undulating relief
Roraima				
Municipality	Rorainópolis		Caracaraí	
Dominant Structural Province	Amazônia		Amazônia	
Dominant Structural Subprovince	Parima-Tapajós		Parima-Tapajós	

Geological Era of Formation	Proterozoic Eon, Paleoproterozoic Era, Orosirian Period	Proterozoic Eon, Paleoproterozoic Era, Orosirian Period
Dominant Lithologies	Granite, monzogranite, monzonite, quartzolite, and syenogranite.	Granite, monzogranite, monzonite, quartzolite, and syenogranite.
Soil Type	PAd - Argissolo Amarelo Distrófico	GXbd - Gleissolo Háplico Tb Distrófico
Texture	medium/clayey	sandy/medium
Relief Type	Gently wavy	Flat
Rondônia		
Municipality	Cadeias do Jamari - FNJD	Itapuã do Oeste - FNJM
Dominant Structural Province	Cenozoic Sedimentary Cover	Amazônia
Dominant Structural Subprovince	Undifferentiated Cenozoic Sedimentary Cover	Rio Negro-Juruena
Geological Era of Formation	Phanerozoic Eon, Cenozoic Era, Quaternary Period, Upper Pleistocene Epoch	Neoproterozoic Era, Tonian Period | Proterozoic Eon, Paleoproterozoic Era, Statherian Period

Dominant Lithologies	clay; sand, and laterite	Gneiss, migmatite, granite, granulite, leptite, charnockite, syenogranite, aplite syenite, orthogneiss, paragneiss, metamafic rocks, calc-silicate rocks, metagranite, metamonzogranite, metapelite, metagabbro, metadiabase, amphibolite, metatonalite, kinzigite, schist, quartzite, and rhyolite.
Soil Type	LAd - Latossolo Amarelo Distrófico	LAd - Latossolo Amarelo Distrófico
Texture	very clayey and clayey	very clayey and clayey
Relief Type	Flat and gently undulating	Flat and gently undulating

UPA: Annual Production Unit. Source: Environmental Information Database. Accessed from https://bdiaweb.ibge.gov.br/#/consulta/geologia.

Table S3. Predominant vegetation types in municipalities where *Manilkara huberi* samples were collected.

			Pará		
Municipality	Juruti	Aveiro UPA 1	Aveiro UPA 10	Belterra km 140	Belterra km 117
Type	Lowland Dense Ombrophilous Forest	Lowland Dense Ombrophilous Forest	Lowland Dense Ombrophilous Forest	Lowland Dense Ombrophilous Forest	Lowland Dense Ombrophilous Forest
			Amazonas		
Municipality	Humaitá	Itapiranga	Parintins		Silves
Type	Open Alluvial Ombrophilous Forest (Floodplain Forest)	Lowland Dense Ombrophilous Forest	Lowland Dense Ombrophilous Forest	Lowland Dense Ombrophilous Forest	Lowland Dense Ombrophilous Forest

Roraima		
Municipality	Rorainópolis	Caracaraí
Type	Submontane Dense Ombrophilous Forest	Submontane Dense Ombrophilous Forest

Rondônia		
Municipality	Cadeias do Jamari<br>FNJD	Itapuã do Oeste<br>FNJM
Type	Lowland Open Ombrophilous Forest	Submontane Open Forest

UPA: Annual Production Unit. Source: Environmental Information Database. Accessed from https://bdiaweb.ibge.gov.br/#/consulta/vegetacao.

Figure S1. Instrument configuration and time-event sequence employed in the EA–IRMS system for gas separation. The figure illustrates the Isodat method, including gas switching (CO_2 and SO_2), reference gas pulses, and gas chromatography (GC) parameters. Chromatographic separation was achieved using a temperature-programmed GC oven, initially set at 70 °C for 250 s, ramped to 240 °C, and held at 240 °C for 500 s, under a continuous flow of high-purity helium (He 5.0).

Isodat Workspace - [3_Triple_N2_CO2-94%_SO2]

File Help

New | Open | Save | Print | Options | Help | States | Windows | Editors

Instrument | Time Events | Compound Names | Evaluation@N2 | Peak Detection@N2 | Printout@N2 | Evaluation@CO2 | Peak Detection@CO2 | Printout@CO2 | Evaluation@SO2 | Peak Detection@SO2 | Printout@SO2

Time [s]	Reference Automatic	GC - Start	Start Flash	Switch Gas	MS Capillary - On
10	🟢				
30	🔴				
50	🟢				
70	🔴				
75			🟢		
150				CO2	
250		🟢			
350	🟢				
370	🔴				
390	🟢				
410	🔴				
415				SO2	
500		🔴			
560	🟢				
580	🔴				
600	🟢				
620	🔴				

Flash IRMS NCS

Conflo Calculation - Path not found : C:\Thermo\Isodat NT\global\User\ConFlo IV Interface\EA IsoLink Device\Databases\

Figure S2. Representative Isodat 3.0.0.83 interface illustrating data acquisition and processing in the EA–IRMS system through the ConFlo IV interface, including the visualization of chromatographic peaks and the determination of isotopic ratios ($\delta^{13}C$ and $\delta^{15}N$).

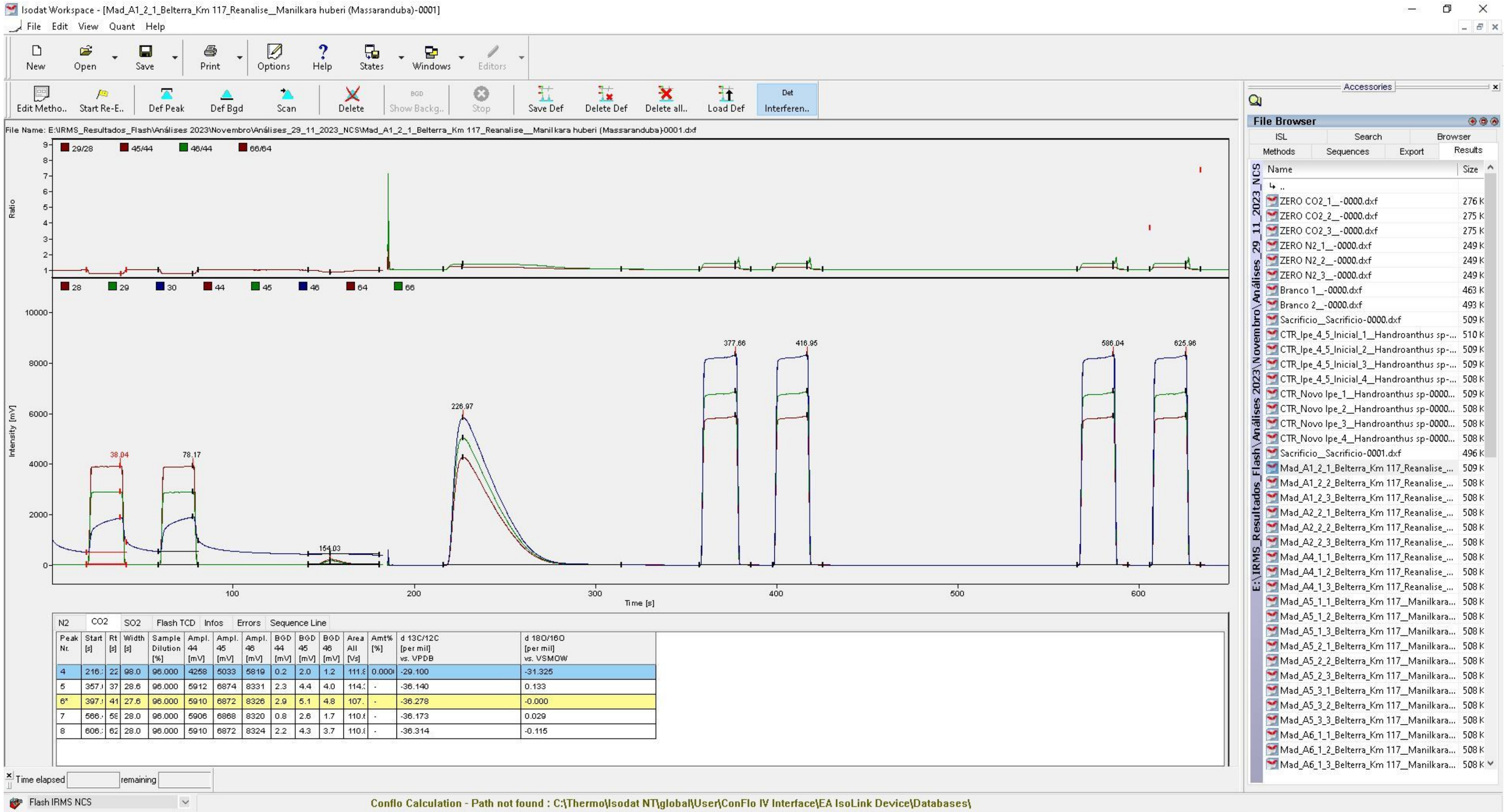

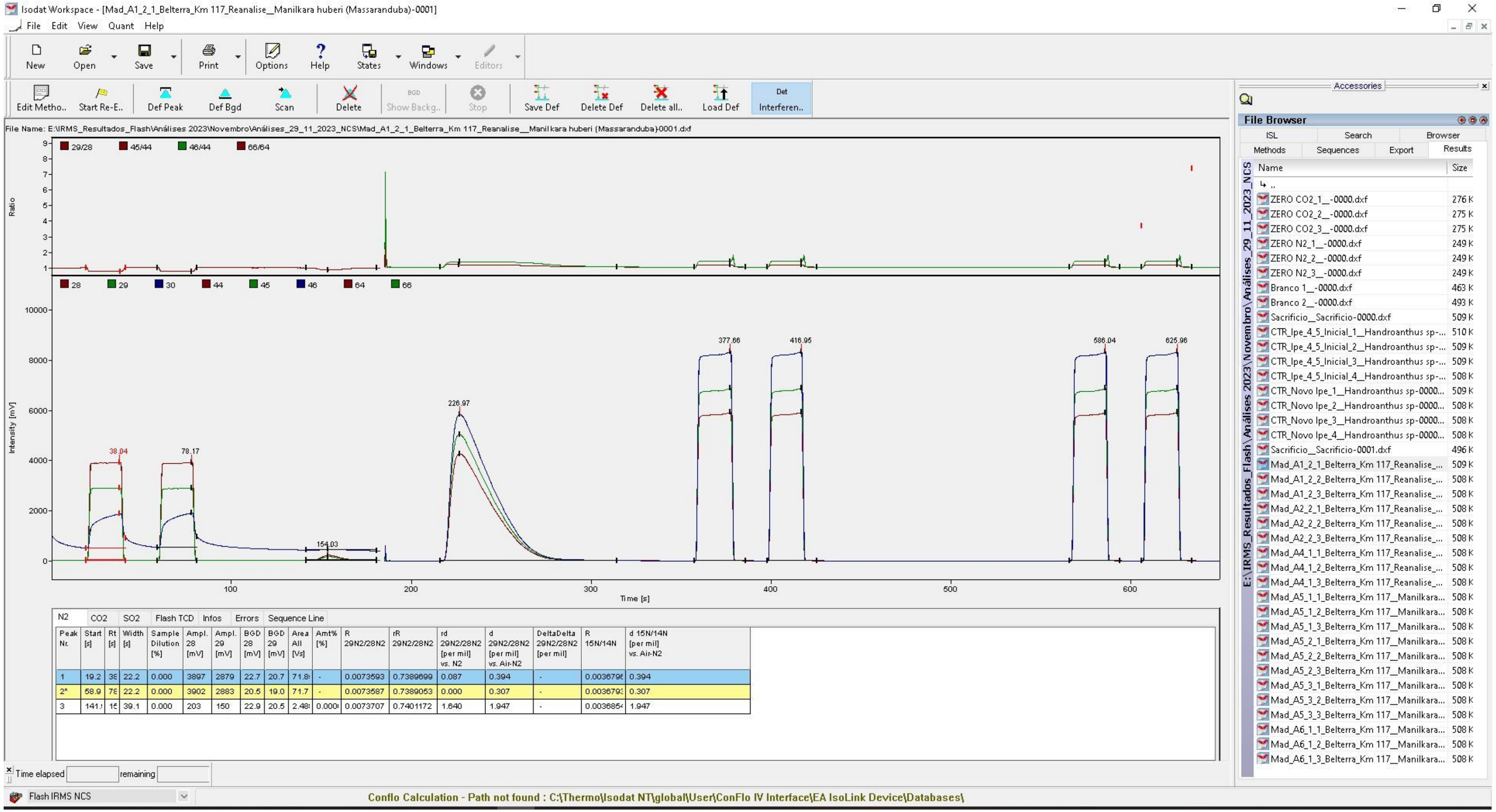

Table S4. Leave-One-Tree-Out (LOTO) Validation of the Linear Discriminant Analysis

Validation procedure	Correct classifications	Total observations	Accuracy (%)
Observation-level LOOCV	102	339	30.09

Validation procedure	Correct classifications	Total observations	Accuracy (%)
Leave-One-Tree-Out (LOTO)	93	339	27.43
Difference	−9	339	−2.66 percentage points
